# Role of MELD Score and Serum Creatinine as Prognostic Tools for the Development of Acute Kidney Injury after Liver Transplantation

**DOI:** 10.1371/journal.pone.0064089

**Published:** 2013-05-23

**Authors:** Thiago Gomes Romano, Ivana Schmidtbauer, Fernanda Maria de Queiroz Silva, Carlos Eduardo Pompilio, Luiz Augusto Carneiro D'Albuquerque, Etienne Macedo

**Affiliations:** 1 ABC Medical School, Nephrology Department, Sao Bernardo, Sao Paulo, Brazil; 2 University of Sao Paulo, Surgical and Intensive Care Unit, Sao Paulo, Sao Paulo, Brazil; 3 University of Sao Paulo, Gastroenterology Department, Sao Paulo, Sao Paulo, Brazil; 4 University of Sao Paulo, Nephrology Department, Sao Paulo, Sao Paulo, Brazil; 5 Hospital Sirio Libanes, Intensive Care Unit, Sao Paulo, Sao Paulo, Brazil; University of Colorado School of Medicine, United States of America

## Abstract

**Background:**

The role of the Model for End-Stage Liver Disease (MELD) score in predicting complications, such as Acute Kidney Injury (AKI), after orthotopic liver transplantation (OLT) has yet to be evaluated and serum creatinine may be too heavily weighted in the existing MELD formula, since it has many pitfalls in cirrhotic patients.

**Methods:**

Retrospective data of the perioperative period from consecutive adult OLTs performed from January to December 2009 were recorded. Univariate and multivariate analysis were performed to analyze the risk factors for AKI and mortality after OLT.

**Results:**

There were 114 OLTs performed in the study period, 22 (19,2%) were submitted to dialysis prior OLT and were excluded from the analysis for AKI. The median age was 52 years and 66% were male. Median creatinine value was 0.85mg/dL and MELD was 19. Fifty-two of the 92 patients (56,5%) developed AKI in the first 72 hours after OLT. The only independent risk factor for AKI was calculated MELD and when the components of the MELD score were analyzed, INR had a much stronger impact in predicting AKI then serum creatinine. Overall mortality rate was 32,5% and anesthesia duration was the only variable associated with higher mortality rate.

**Conclusions:**

Although MELD score seems to have a good performance in predicting AKI after OLT, serum creatinine had no impact on its prediction despite its importance on MELD calculation. Modifying the MELD score, which could include novel AKI biomarkers, may improve its prognostic accuracy and provide a better tool for public health planning.

## Introduction

Understanding the prognosis of the cirrhotic patients awaiting orthotopic liver transplantation (OLT) and assessing morbid-mortality risk factors are critical points in the development of strategies to improve long-term outcomes and assist in planning public health strategies.

For over a decade, the Model for End-Stage Liver Disease (MELD) has been used worldwide as the criteria for organ allocation in patients with chronic liver disease [1]. MELD has been a useful tool to predict mortality for patients awaiting OLT. However, the role of the score in predicting complications after OLT has yet to be evaluated [2]; [3].

The MELD score for prioritizing organs for liver transplantation is calculated based on serum creatinine, the international normalized ratio (INR) and serum bilirubin. Renal dysfunction, measured by the pre-procedure serum creatinine, is a key component of that score [4]. However, although pre-procedure serum creatinine is a well recognized risk factor for Acute Kidney Injury (AKI) in many surgical scenarios [5]; [6], some studies do not confirm this correlation in liver transplanted patients [7]; [8]. Despite the inaccuracy of creatinine as a marker for renal dysfunction in cirrhotic patients, it is still used as a gold standard for the diagnosis of AKI after OLT.

AKI after OLT is a common complication, with incidences ranging from 12–95%[9]–[13]. Deterioration in renal function in this setting is also associated with increased 30-day mortality rate, graft dysfunction and 1-year mortality [2]; [14]. In the last decade, there have been many efforts to improve perioperative management and to enhance the use of intervention drugs with less nephrotoxicity [15]. Still, there remains a lack of understanding about the risk factors leading to AKI after OLT.

In this study, we aim to evaluate the role of the MELD score and pre-procedure serum creatinine in predicting AKI after OLT.

## Methods

The Ethical Committee of Hospital das Clinicas of Sao Paulo Medical School approved this study. The Ethical Committe stated that written consent was not needed since data collected did not identify patients, and there was no intervention propsed by the researchers.

We retrospectively reviewed the medical records of consecutive adult OLTs performed from January to December of 2009.

Information recorded from patients’ charts included demographic characteristics: age, gender, previous history of hypertension (HTN) and diabetes mellitus (DM). Preoperative laboratory values were also recorded: reference creatinine, INR, bilirubin, infection prior surgery, MELD value of indications of OLT (taking into account the presence of factors such as hepatocellular carcinoma, hepatopulmonary hypertension and neuroendocrine metastatic tumor, as described in the Brazilian National Transplant System legislation) and calculated MELD.

From the intraoperative period, we recorded use of terlipressin, volume of blood components transfused, fluid balance, duration of anesthesia, maximum pulmonary artery pressure and maximum cardiac index. Post operative factors recorded included: maximum lactate levels during the first 24 hours after ICU admission, noradrenaline dose of admission in ICU and need for re-transplantation. We also noted the days of in-hospital stay before transplant, donor age and height, as well as in-hospital mortality rates.

AKI was defined as an increase equal or greater than 0.3 mg/dL in serum creatinine in the first 72 hours after procedure. Reference creatinine was defined as the last creatinine available before the transplantation procedure, measured by the colorimetric kinetic method. The diagnosis of AKI in our study was based on only one of the components of the Acute Kidney Injury Network (AKIN) [16] since data on urinary output was not available.

### Statistical Analysis

Variables in the study were evaluated by the Shapiro-Wilk W test and distribution plots to test normality of distribution. Data that did not meet normality assumptions are presented as median and percentiles [P25^th^/P75^th^] and the Mann-Whitney U test was used to compare groups. For categorical variables, the Pearson χ^2^ test or Fisher’s exact test was applied as appropriate. We evaluated the association between baseline characteristics and perioperative factors with the development of AKI within 72 hours after transplant. We excluded patients who needed renal replacement therapy (RRT) before transplant from the analysis of the risk factors for AKI. The risk factors for in-hospital mortality were analysed, including development of AKI and need for RRT 72 hours after OLT.

Multivariate logistic regression analysis was used to evaluate variables that were independently associated with development of AKI and mortality. Predictors related to AKI or mortality on univariate analysis (p<0.10) were entered into model. A backward stepwise elimination algorithm was used with a p<0.05 for predictors to remain in the final model.

A Receiver-Operating Curve (ROC) was performed to examine the discriminating power of MELD for the outcome AKI.

Statistical analyses were performed with SPSS 19 software.

## Results

There were 114 patients submitted to OLT in the study period. Twenty-two patients needed RRT before transplant and were excluded from the analysis of the risk factors for AKI but included in the analysis for mortality. Of the 92 patients analyzed, the median age was 52 (40.5–59) years old. Most of the patients were male (66%), the incidence of hypertension (HTN) was 14% and diabetes mellitus (DM) 24%. The median reference creatinine was 0.85 mg/dL (0.66–1,22 mg/dL) and median calculated MELD was 19 (10–29) (Table 1).

**Table 1:** Baseline characteristics of study population.

Characteristics	All (92)
Age (years)	52 (40.5–59)
Gender (♂)	61/92
HTN	13/92
DM	22/92
Reference Creatinine (mg/dL)	0.85 (0.66–1.22)
MELD of indication	29 (23–29)
Calculated MELD	19 (10–29)
Infection prior surgery	19/92
INR	1.5 (1.2–2.15)
Bilirubin (mg/dL)	3.0 (0.95–6.03)
In- hospital days prior surgery	1 (1–2)

Fifty-two of the 92 patients (56.5%) developed AKI in the first 72 hours period after OLT. Table 2 shows the risk factors associated with AKI. Patients who developed AKI had significantly higher bilirubin, INR, MELD of indication, calculated MELD, use of terlipressin during surgery, volume of blood components administrated and dose of noradrenaline at ICU admission.

**Table 2:** Risk factors for the development of AKI.

Characteristics	All 92	AKI 52 (56.5%)	Non AKI 40 (43.5%)	P
Age (yr)	52 (40.5–59)	51.5 (40.5–59)	52 (36–57.5)	0.81
Gender (♂)	61/92	36 (59%)	25 (41%)	0.49
HTN	13/92	5 (38.5%)	8 (61.5%)	0.15
DM	22/92	11 (50%)	11 (50%)	0.48
Reference creatinine (mg/dL)	0.85 (0.66–1.22)	0.92 (0.76–1.36)	0.83 (0.67–1.11)	0.21
Bilirubin	3.0 (0.95–6.03)	4.5 (2.8–9.6)	1.9 (0.8–3.7)	0.001*
INR	1.5 (1.2–2.15)	1.9 (1.5–2.7)	1.25 (1.12–1.57)	0.000*
In-hospital days prior surgery	1 (1–2)	1 (1–3)	1(1–1)	0.002*
Infection prior surgery	19/92	15 (79%)	4 (21%)	0.02*
MELD of indication	29 (23.5–30.5)	29 (24–31.5)	29 (20.5–29)	0.042*
Calculated MELD	21 (11.5–31)	25.5 (17–32)	11 (8.5–18.5)	<0.001*
Donor age (yr)	41.5 (31–55)	45 (30–60)	39 (32–53)	0.63
Donor Height (Kg)	75 (65–80)	70 (65–80)	79 (65–85)	0.16
Use of terlipressinduring surgery	41/92	30 (73.2%)	11 (26.8%)	0.003*
Volume of blood components (ml)during surgery	790 (305–1482)	830 (460–1480)	367 (145–927)	0.003*
Fluid balance (L)	5.4 (4.0–7.0)	5.6 (4.1–7.1)	5 (3.3–6.8)	0.13
Anesthesia duration (h)	9 (8–10.15)	9.0 (8–10.1)	8.3 (7.2–10)	0.28
Maximun PAP (mmHg)	24 (20–28)	24 (21–28)	22 (20–26.5)	0.29
Maximum cardiac index (L/min/m^2^)	5.5 (4.5–6.4)	5.5 (4.7–6.8)	5.5 (4.5–6.2)	0.42
Maximum lactate levels during the first24 hours after ICU admission (mg/dL)	49 (34.5–80.5)	52 (36–97)	49 (30–59)	0.07
Noradrenaline dose of admission in ICU(mcg/Kg/min)	0.1 (0.02–0.2)	0.14 (0.05–0,2)	0.05 (0.0–0.16)	0.01*
Need for retransplantation	4/92	3 (75%)	1 (25%)	0.43

**Figure 1:**
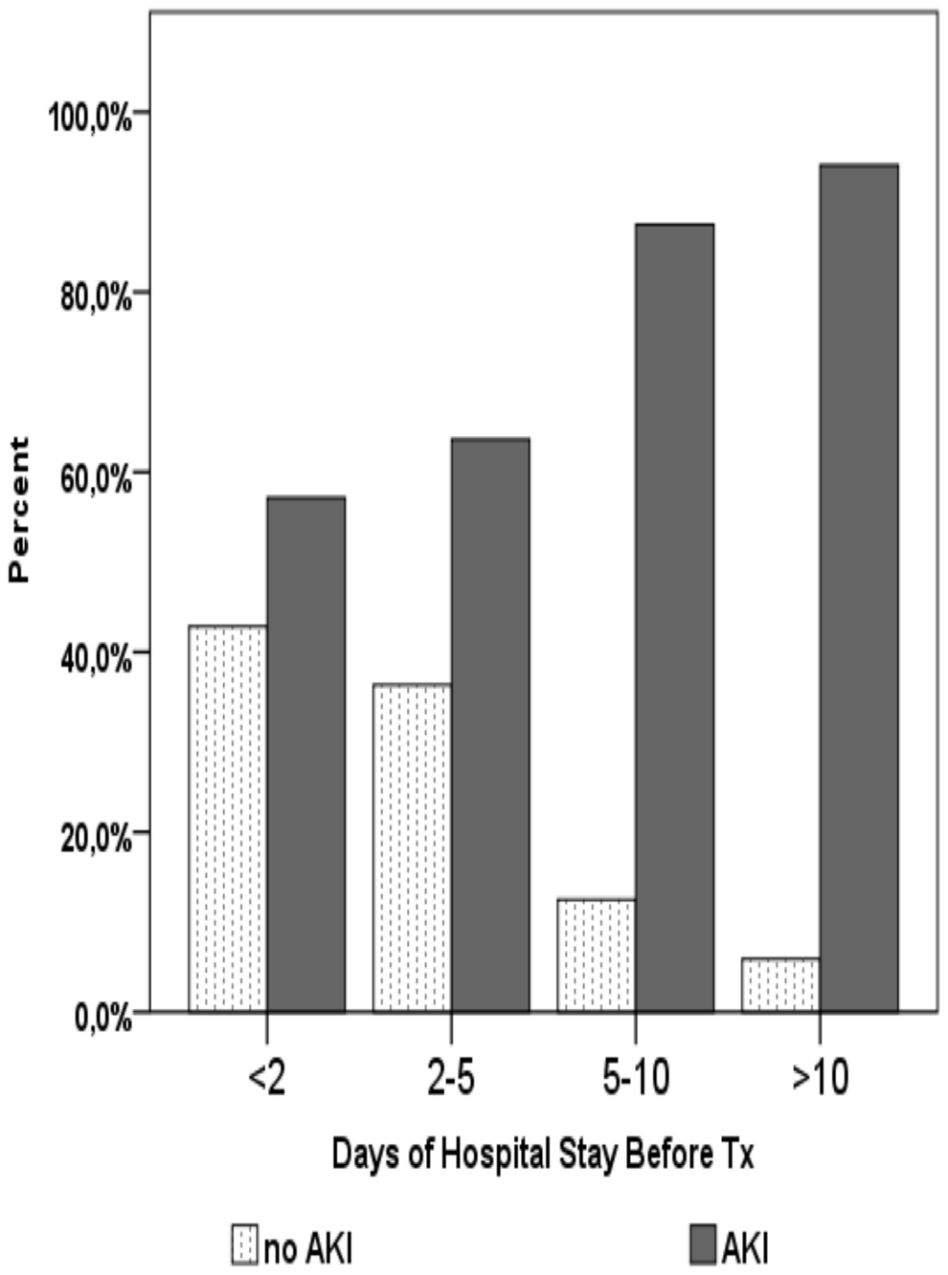
Days of in-hospital stay prior OLT and AKI. OLT: Orthotopic liver transplant; AKI: Acute Kidney Injury; Tx: Transplant.

Days of in-hospital stay prior to surgery and infection prior to surgery were also associated with development of AKI. Patients with more than two days of in-hospital stay prior to surgery had an incidence of AKI above 60% after OLT (figure 1). Those with more than 5 days had an AKI incidence of 83%.

**Figure 2:**
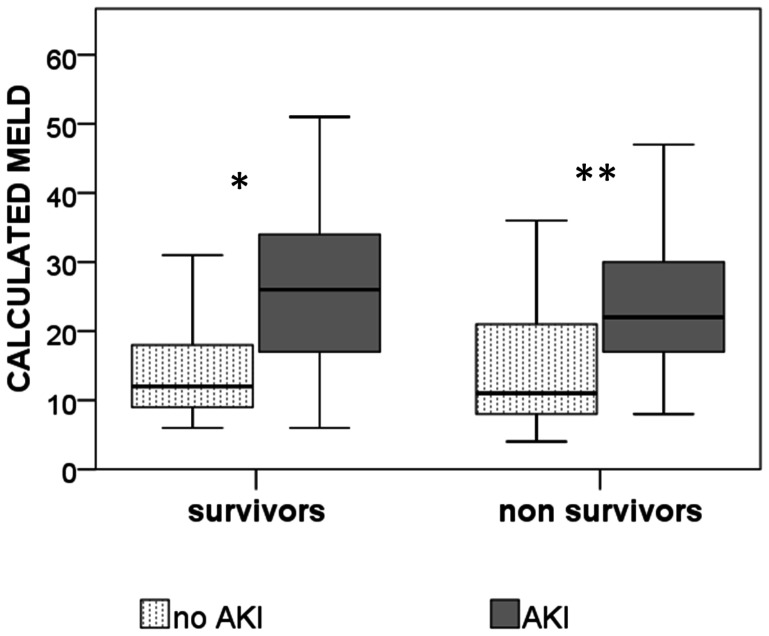
Calculated MELD and incidence of AKI in survivors and non survivors. *p = 0.002; **p = 0.41. MELD: Model for End-Stage Liver Disease; AKI: Acute Kidney Injury.

The MELD score was also associated with AKI. Higher scores were associated with higher incidence of AKI in survivors (figure 2). In the multivariate analyses, calculated MELD was the only variable associated with AKI after OLT (table 3). When the components of the MELD score were analyzed, INR compared to creatinine was a much stronger factor associated with AKI (table 4). Reference creatinine was not associated with either mortality or AKI in our population, despite its high impact on MELD calculation.

**Table 3:** Multivariate analyses for AKI.

Characteristics	Odds ratio	p value	Coefficient (95% Confidence Interval)
Infection prior surgery	3.4	0.19	0.54–21.86
Calculated MELD	1.03	0.04*	1.00–1.06
In-hospital days prior surgery	0.95	0.61	0.81–1.13

Hosmer and Lemeshow test: 0.34.

**Table 4:** Analyses of the components of MELD × AKI.

Characteristics	Odds Ratio	p value	Coefficient (95% Confidence Interval)
Reference creatinine	0.42	0.10	0.14–1.19
INR	2.01	0.03*	1.06–3.82

Hosmer and Lemeshow test: 0.67.

**Figure 3:**
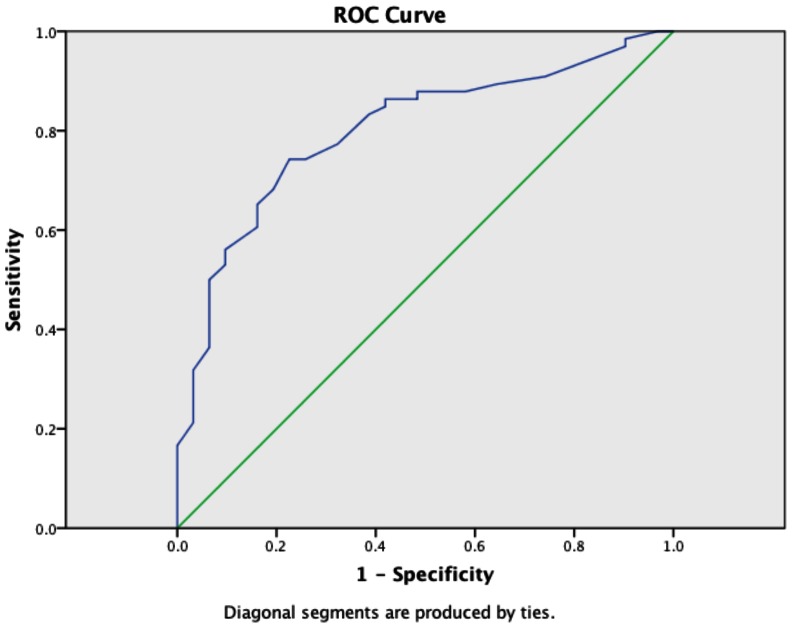
ROC curve of MELD versus AKI. Area Under Curve (AUC): 0.8. ROC: Receiver-Operating Curve; MELD: Model for End-Stage Liver Disease; AKI: Acute Kidney Injury.

The ROC curve of MELD for predicting AKI demonstrated an area under curve (AUC) of 0.8 as shown in figure 3. The optimal MELD score for sensibility and specificity in this context was 18.

Overall in-hospital mortality rate was 32.5%. Mortality rate of patients developing AKI after surgery was 38.5%. The factors associated with the risk of death were maximum lactate levels during the first 24 hours of intensive care unit (ICU) admission, need for re-transplant and need for renal replacement therapy (RRT), as shown in table 5.

**Table 5:** Univariate analyses for mortality.

Characteristics	All 114	Non survivors 37 (32.5%)	Survivors 77 (67.5%)	P
Age (yr)	52 (40.5–59)	53 (40.5–61)	52 (40.5–57.5)	0.36
Gender (♂)	74/114	24 (32.4%)	50 (67.6%)	0.99
HTN	19/114	6 (31.6%)	13 (68.4%)	0.92
DM	27/114	9 (33.3%)	18 (66.7%)	0.91
Reference creatinine (mg/dL)*	0.87 (0.72–1.23)	0.93 (0.81–1.41)	0.83 (0.69–1.18)	0.30
MELD of indication	29 (23.5–30.5)	27 (21–34)	29 (24–30)	0.71
Calculated MELD	21 (11.5–31)	24 (10.5–34)	20 (12.25–30.75)	0.54
In-hospital days prior surgery	1 (1–5)	1 (1–5.5)	1 (1–5)	0.99
Need for dialysis prior transplantation	22/114	7 (31.8%)	15 (68,2%)	0.94
Infection prior surgery	34/114	14 (41.2%)	20 (58.8%)	0.2
Donor age (yr)	40.5 (30–55.7)	49.5 (34–63.5)	38.5 (29.2–52.75)	0.08
Donor Height (Kg)	75 (65–80)	75 (63.5–80)	74 (65–80)	0.96
Use of terlipressin	51/114	20 (39.2%)	31 (60.8%)	0.17
Volume of blood components (ml)During surgery	790 (305–1482)	927.5 (600–1550)	694 (227.5–1422.5)	0.14
Fluid Balance (L) During surgery	5.5 (4.0–7.2)	5500 (4300–8050)	5400 (3900–7000)	0.40
Anesthesia Duration (h)	9 (8–10.15)	9.3 (8–11.7)	8.3 (7.42–10)	0.07
Maximun PAP (mmHg) during surgery	24 (20–28)	24.5 (19.5–28)	24 (20.75–28)	0.68
Maximum cardiac index (L/min/m^2^)during surgery	5.6 (4.6–6.5)	5.2 (4.5–6.6)	5.8 (4.65–6.55)	0.34
Post-operative AKI	52/92	20 (38.5)	10 (25)	0,17
Dialysis (72 h)	13/114	8 (61.5%)	5 (38.5%)	0.02*
Need for readmission	74/91	21 (28.4%)	53 (71.6%)	0.31
Need for retransplantation	4/114	4 (100%)	-	0.02*
Maximum lactate levels during the first24 hours after ICU admission (mg/dL)	48 (31–78.5)	76 (34.2–115.5)	45 (30–58.75)	0.005*
Noradrenaline dose of admission in ICU(mcg/Kg/min)	0.1 (0.03–0.2)	0.12 (0.03–0.5)	0.1 (0.02–0.2)	0.12

* from those patients free from dialysis prior OLT.

After multivariate analyses, anesthesia duration was the only variable associated with higher mortality rate (Table 6).

**Table 6:** Multivariate analyses for mortality.

Characteristics	Odds ratio	p value	Coefficient (95% Confidence Interval)
Anesthesia duration	0.89	0.001*	0.82–0.95
Volume of blood components (ml) Duringsurgery	1.00	0.14	1–1.001
Infection prior surgery	1.16	0.73	0.48–2.80
Use of terlipressin	1.25	0.58	0.55–2.86

# Hosmer and Lemeshow test: 0.83.

## Discussion

Cirrhotic patients have a high risk for surgical and clinical complications after OLT. The number of patients on waiting lists for OLT far outnumbers available grafts [17]. In this scenario, the development of prognostic tools after OLT is of critical importance. These tools can help to design better health care strategies and inform decision-making by the potential transplant recipients and their relatives [18].

One of the early complications with prognostic relevance after OLT is AKI. The development of AKI is multifactorial, resulting from pre, intra and postoperative factors. In our study the preoperative conditions of the patient were the most important predicting factors for the development of AKI. The bilirubin, INR levels, MELD of indication, calculated MELD, in-hospital days prior surgery and the presence of infection prior surgery were the factors associated with AKI.

Despite the importance of pre-procedure clinical status, which will be discussed further, intraoperative hemodynamic management is essential for kidney protection since decline in systemic vascular resistance (SVR) and mean arterial pressure (MAP) are markers of reperfusion phase. The post reperfusion syndrome (PRS) is known to be determinant for the development of AKI, as demonstrated by Paugam-Burtz et al. In this study, 70% of the patients whot had 30% reduction in MAP during reperfusion phase developed severe renal dysfunction [19]. In addition, the control of intraoperative hypotension often requires the use of large volume of plasma expanders, blood components and drugs such as phenylephrine, noradrenaline and terlipressin. In our study we observed a positive relationship between terlipressin use, blood components transfusion during surgery, dose of norepinephrine in ICU admission and the development of AKI.

When analyzing the postoperative factors, there is significant evidence for the role of immunosuppression drugs such as calcineurin inhibitors in renal dysfunction after OLT [20]. However, this discussion is not within the scope of this paper; here, we address the factors related mainly to pre and intraoperative period.

The data from the preoperative management has changed since the implementation of the MELD score in the United States in 2001. Many centers have shown reduction of mortality rates for liver transplant candidates on the waiting list [21]. The score gives priority to candidates with more severe disease. Therefore, OLT tends to be performed on sicker patients with shorter waiting times [22].

The role of the MELD score in predicting mortality in cirrhotic patients seems to be well established, but its ability in predicting post-liver transplantation survival and prognosis is questionable. Although pre-procedure serum creatinine level seems to be an effective prognostic tool in many surgical scenarios, its use as a prediction of AKI in cirrhotic patients is debatable [23]. Sharma et al. has proposed that serum creatinine may be too heavily weighted in the existing MELD formula [24]. Another pitfall suggested by the same author is that the score does not distinguish creatinine levels within the range of 0 to 1 mg/dL. Therefore, all the patients with serum creatinine below 1 mg/dL have the same grade in this criterion of the score.

Creatinine–based formulas such as Cockcroft and Modification of Diet in Renal Disease (MDRD) are also inaccurate in cirrhotic patients [25]. This may be because protein-calorie malnutrition is common in this setting [26]. Another problem is that elevated serum bilirubin can interfere with the measurement of serum creatinine when the colorimetric Jaffe method is used rather than an enzymatic one. In this scenario, the glomerular filtration rate (GFR) is overestimated using creatinine-based formulas.

Francoz et al., in a well design study, compared the GFR measured by iohexol clearance with creatinine-based equations in pre OLT patients. They demonstrated that MDRD and Cockcroft formulas overestimate by 20% the GFR in 43% of the cases, and that body mass index (BMI) was one of the factors associated with this overestimation. In the same study, a score based on true GFR had a better accuracy for predicting mortality than MELD score. They proposed that a score including true GFR instead of serum creatinine would be more accurate in predicting early mortality in patients with cirrhosis awaiting for OLT [27]. Recently Wagner et al. demonstrated that cystatin C based equations were superior than creatinine for predicting renal dysfunction after OLT [28].

This evidence suggests a necessity for the improvement of the MELD score. Kim et al. studied the role of the score, serum sodium concentration and the interaction between the two in predicting mortality among patients on a waiting list for liver transplantation and found that serum sodium was associated with a higher risk of mortality independent of the MELD score. This effect was greater in patients with lower MELD score. From this data, the author proposed a new calculation of the score, adding the value of serum sodium. The difference between the so called MELDNa and MELD was large enough to affect allocation priority. About 7% of the patients who died on the waiting list could have been transplanted and death prevented when using MELDNa [29]. In the United Kingdom, a new scoring system was incorporated in 2008, with the ultimate goal of developing a better scoring system by creating an allocation model that takes into account the net benefit of transplant. This score is derived from patient’s serum sodium, creatinine, bilirubin and INR. Using the UKELD score, a 1-year mortality rate without transplantation greater than 9% is the minimal listing criteria [30].

We focused our study on the possibility of improvement of MELD score, especially in the renal function component and its ability to predict postoperative complications. One of the most important findings in our study is the absence of correspondence between MELD score and serum creatinine value in predicting AKI after OLT. Weigand et at. also demonstrated that serum creatinine at the time of OLT was not related to the need of RRT after 3 months of the transplant procedure [31].

Since creatinine value is a key component of the score calculation, it can be assumed that MELD score and serum creatinine are both associated with the risk to develop AKI. However, we found that although MELD had a good performance in predicting AKI, pre-procedure serum creatinine did not. Interestingly, when the components of MELD score were analyzed individually, INR was the strongest factor associated with AKI. In the multivariable analyses, calculated MELD was the only variable that had an impact on AKI development, with a good area under the ROC curve (0.8).

Our study has limitations that need to be taken into account. The first is that it is a retrospective single center study in which the data was obtained from medical records. Secondly, renal function was monitored only on serum creatinine levels, because we did not have the data of urine output from the patients’ records. We defined AKI by the rise on serum creatinine levels by 0.3 mg/dL during the first 72 hours after OLT. This decision is not in accord with the criteria from the AKIN [16] and most recently the Kidney Disease Improving Global Outcomes (KDIGO) [32] where AKI is defined by the 0.3 mg/dL raise on serum creatinine during the first 48 hours or 50% decline in glomerular filtration rate during the next 7 days. The third limitation that is worth noting is that we proposed and found that pre operative serum creatinine is not a risk factor for AKI in OLT, but we still used the serum creatinine levels as the standard for the diagnosis of AKI in our population.

### Conclusion

The results of our analyses demonstrate that the development of AKI after OLT is strongly associated with hepatic function tests rather than serum creatinine. This reinforces the need for other kidney function assays in cirrhotic patients and challenges the value of serum creatinine in MELD calculation within the OLT population. Although MELD score has a good performance in predicting AKI after OLT, serum creatinine had no impact as a prognostic tool for AKI in OLT patients, despite its high impact on MELD calculation. Variables that denote liver function, such as INR, seem to be better predictors of AKI after the first 72 hours of transplantation.

These data support the evidence that better AKI biomarkers are needed in the liver transplant scenario. Modifying the MELD score, which could include those biomarkers, may improve its prognostic accuracy after OLT and provide a better tool for public health planning.
